# Voters’ Attributions of Psychopathic Traits to Donald Trump and Hillary Clinton After the 2016 U.S. Presidential Election and the 2020 Trump Impeachment Trial

**DOI:** 10.5964/ejop.14475

**Published:** 2025-05-28

**Authors:** Paul E. Jose, Ira J. Roseman, Anna Geiserman, Taylor Winter, Boris Bizumic

**Affiliations:** 1 Victoria University of Wellington, Wellington, New Zealand; 2 Rutgers University Camden, Camden, NJ, USA; 3 Australian National University, Canberra, Australia; Victoria University of Wellington, Wellington, New Zealand

**Keywords:** psychopathy, authoritarianism, mental health literacy, politics, voters

## Abstract

We sought to determine if voters’ personological characteristics influence perceptions of psychopathic traits in political candidates and predict vote choice. Our first dataset was collected soon after the 2016 U.S. presidential election between Hillary Clinton and Donald Trump. The sample, 159 Trump voters and 154 Clinton voters, rated their own authoritarian beliefs and their perceptions of psychopathic tendencies in both candidates, and reported their vote. As predicted, Clinton voters perceived low levels of psychopathic tendencies in Clinton and high levels of psychopathic tendencies in Trump, and Trump voters displayed the opposite pattern. A concurrent mediation analysis found that highly authoritarian voters perceived Trump to be low on psychopathic tendencies, and they tended to vote for Trump. These results were replicated from a different sample of 300 voters about three years later, soon after Trump’s first impeachment. The results suggest that authoritarian beliefs profoundly color perceptions of psychopathy in political candidates.

The U.S. presidential election of 2016 was widely perceived by political experts as an anomalous event (e.g., Gibson & Shaw, 2019), with one candidate presenting traits outside the expected norms. One candidate, Donald Trump, gained support during the primaries as an “outsider” who seemed to flout traditional rules of political campaigning, that is, he rejected “political correctness” and overtly embraced polarizing political and social positions. Political commentators, particularly left-leaning ones, dismissed his candidacy as amateurish, off-putting, and divisive. Seventy-five experts in U.S. politics described Trump’s personality and campaign style several weeks after the 2016 election (Nai & Maier, 2018) as “populist, negative, and based on fear appeals” (p. 80). Notably, relevant to the present research efforts reported here, Trump was perceived by these experts as exhibiting higher levels of psychopathic behaviors (e.g., ‘shows a lack of remorse’). Nevertheless, Donald Trump won the GOP presidential nomination and the Electoral College, despite losing to Hillary Clinton in the popular election by almost 3 million votes.

Political scientists and social and personality psychologists continue to assess the shockwave of this political event. As part of this endeavor, the present study was conducted to examine how laypeople’s background personological traits affect perceptions of candidates. We know that knowledge bases and belief systems (e.g., evangelical Christian beliefs) affect perceptions of candidates and thus affect vote choice (e.g., Layman, 1997). The intent of the present study was to extend this insight to authoritarianism. The study of authoritarian beliefs within politics has a long history, dating back to World War II (Gøtzsche-Astrup & Hogg, 2024), and a fruitful line of research has demonstrated that this psychological construct significantly shapes voting behavior (Conway & McFarland, 2019; Godbole et al., 2022; Ludeke et al., 2018). Further, we wanted to investigate whether authoritarian beliefs of a voter affect their perceptions of psychopathic characteristics in the candidates and thereby influence for whom they vote.

## How Accurately Can Laypeople Detect Mental Illness in Others?

Is the average citizen equipped with the requisite knowledge and person perception skills to discern whether or to what degree a political candidate possesses disqualifying psychiatric traits? Jorm (2000) has referred to this knowledge base as *mental health literacy*, and a number of studies have been devoted to assessing the extent to which non-experts can accurately label vignettes of hypothetical exemplars of certain types of mental illness. Jorm et al. (1997) reported that 39% of Australian adults correctly labelled an instance of depression and only 27% correctly labelled an instance of schizophrenia. Regier et al. (1988) demonstrated that American non-experts were also more familiar with negative mood disorders than other types of mental illness but generally were not very accurate. Furnham et al. (2011) have noted that British university students had “heard of” just over one-third of 90 psychiatric illnesses. Lam (2014) and Vovou et al. (2021) have shown that mental health literacy varies for different syndromes, and recognition rates fall below 50% for psychotic syndromes and personality disorders. Few studies have been performed on the ability of laypeople to accurately identify psychopathy, but a study by Furnham et al. (2009) showed that only about 39% of a community sample correctly identified psychopathy compared to 97% for depression and 61% for schizophrenia. Jorm (2000) concluded, “Many members of the public cannot correctly recognize mental disorders and do not understand the meanings of psychiatric terms” (p. 396).

Most studies of mental health literacy have focused on disorders frequently discussed in the media and common human discourse: depression, anxiety, and schizophrenia. However, as noted by Furnham et al. (2011), numerous other psychiatric illnesses are diagnosed and treated by mental health professionals yet are poorly understood by laypeople. Furnham has demonstrated this tendency with regard to narcissistic personality disorder (Wright & Furnham, 2015), borderline personality disorder (Furnham et al., 2015), and psychopathy (Furnham et al., 2009). These mental illnesses fall in the area of *personality disorders*, and the DSM-5 has defined a personality disorder (PD) in this way: “The essential features of a personality disorder are impairments in personality (self and interpersonal) functioning and the presence of pathological personality traits.” Included in this broad category are specific syndromes such as antisocial personality disorder, borderline personality disorder, narcissistic personality disorder, and several other types. Although disagreement exists regarding the precise boundaries among these subtypes of PDs (Anderson & Sellbom, 2018), most psychologists suggest that the construct of *psychopathy*, the topic of the present study, significantly overlaps with antisocial personality disorder. Original measures of psychopathy (e.g., Hare, 1991) were chiefly based on affective and interpersonal traits such as conning behavior and lack of remorse, but subsequent definitions have been more focused on habitual breaching of societal norms and engaging in non-normative behavior (Hare, 1993, 1996). To be consistent with the historical roots of the construct, we base our assessment on Hare’s Psychopathy Checklist–Revised (Hare, 2012), which has been widely adopted as a reliable and valid assessment of psychopathy.

Laypeople tend to be unaware of the features of this psychiatric disorder and often describe psychopaths that they encounter in their everyday life as “manipulative,” “mean,” and “inconsiderate” (Hare, 1999; Stout, 2006). We conducted the present study to examine the degree to which mental health non-experts (in this case, voters) would attribute psychopathic tendencies to political candidates and, further, whether these attributions would be shaped by the voter’s endorsement of authoritarianism.

## Authoritarian Belief System

We sought to investigate one possible basis for diverging opinions about mental health between the two U.S. presidential candidates: *authoritarian beliefs* held by the voter. Authoritarian beliefs have been studied in the U.S. since World War II, and a number of self-report measures have been created to assess the “authoritarian personality” (Adorno et al., 1950), such as the Right-Wing Authoritarian (RWA) Scale (Altemeyer, 2007) and the related measure of beliefs in rigid social hierarchies called the Social Dominance Orientation (SDO) scale (Pratto et al., 1994). Consistent with findings by Crowson and Brandes (2017), we hypothesized that voters reporting high levels of RWA/SDO personality traits would likely vote for Donald Trump and would likely not vote for Hillary Clinton. Trump has been perceived by many American citizens as authoritarian/socially dominant in outlook and behavior (Walt, 2017), whereas the other candidate, Hillary Clinton, in the lead-up to the election accepted the label of being “moderate and center” (Merica, 2015). Further, we reasoned that if a potential voter held strong authoritarian/socially dominant beliefs, they would be less likely to attribute psychopathic traits to Trump, viewing his striking behavior instead in a positive light, that is, believing he was “a strong leader” and “his own man.” In contrast, we predicted that a voter espousing low levels of authoritarianism would be more likely to view Trump’s behavior as evidence of psychopathy, that is, pathological lying, sexual predation, and conscienceless manipulation.

## Predictions of the Present Study

We designed our research efforts to determine how voters attribute psychopathic traits to presidential candidates, which we believe is a novel contribution to the literature in political psychology, although a journalist observed over a decade ago that some politicians exhibit clinical psychopathy (Silver, 2012). First, we sought to determine whether samples of voters, equally distributed between Trump and Clinton voters, would evidence support for a main effect difference in attributed psychopathy between the candidates. Specifically, based on expert ratings of the personalities and behaviors of the two candidates (Nai & Maier, 2018; Visser et al., 2017), we predicted that Trump would be perceived as exhibiting more psychopathic traits than Clinton, although the size of the effect was expected to be small. Second, given that it has been demonstrated that political social identity significantly shapes and molds perceptions of ingroup vs. outgroup members (Basyouni et al., 2022; Sparkman et al., 2019; Wright & Tomlinson, 2018), we also expected to see Trump and Clinton voters diverging in opposite directions in attributing psychopathic tendencies to the two candidates consistent with the “eye of the beholder” theory (Killian, 2023) that voters exhibit (i.e., seeing positive traits in their favored candidate and negative traits in their disfavored candidate). And third and last, we sought to identify associations among the three variables of: (a) background authoritarian beliefs of voters with, (b) attributions of psychopathy symptoms of the two candidates with, (c) the person’s voting choice. In particular, we thought that individuals endorsing high levels of authoritarian/socially dominant beliefs would view Trump as normal (i.e., non-psychopathic) and likely rate Clinton as higher than Trump on an array of psychopathic traits, and these ratings, in turn, would be likely to predict votes for Trump. In contrast, we thought that low authoritarian/socially dominant belief individuals were likely to perceive high psychopathic tendencies in Trump and low psychopathic tendencies in Clinton, and these ratings, in turn, would be likely to predict votes for Clinton.

Our research effort constituted two data collections: (1) Study 1, two weeks after Donald Trump’s inauguration as U.S. president on January 20, of 2017 and, (2) Study 2, shortly after Donald Trump’s acquittal in his first impeachment, on February 5, 2020. The second collection of data (with different participants) was employed in an attempt to replicate Study 1’s basic findings three years later, as well as to determine whether events of the intervening three years might have altered associations identified in Study 1.

## Method: Study 1

### Participants

Using the online survey tool MTurk, we recruited adults who had voted in the 2016 U.S. presidential election. We sought a total sample in excess of 300 to allow the identification of small effect sizes with power at .80 and *p* < .05 (Cohen, 1992). We obtained 159 individuals who reported that they had voted for Donald Trump and 154 individuals who reported that they had voted for Hillary Clinton. Although research indicates that MTurk volunteers are representative of American adults (Levay et al., 2016), a limitation of the study was that we could not determine whether the present sample was representative of all U.S. voters in the 2016 election.

The sample included 182 males and 131 females, and this ratio was similar for Clinton and Trump voters in the sample, χ^2^(1) = .01, *ns*. The age range was 18 to 70 years, and the average age of respondents was 35.04 years (*SD* = 10.6). No difference in age between Clinton and Trump voters was found, *t*(311) = .07, *ns*. In the overall sample, ethnic group identification was weighted toward non-Hispanic Whites (75%) in comparison with African American, Hispanic American, and others (25% total). A contemporaneous accounting of U.S. voters yielded 69% non-Hispanic Whites and 31% Hispanic, Black, and Other registered voters (Gramlich, 2020). In our data, more European Americans than non-European American individuals voted for Trump than for Clinton, χ^2^(1) = 5.65, *p* = .019. Frequencies of the types of highest level of schooling were: high school (12%), some college education (31%), a four-year college degree (40%), and advanced degree (13%). A significant chi-square result was obtained for schooling by endorsed candidate, χ^2^(5) = 11.61, *p* = .04, which was accounted for by more high school graduates and individuals with some college education being counted among Trump voters and more individuals with four-year college degrees among Clinton voters.

### Measures

#### Authoritarian and Socially Dominant Beliefs

The first measure was the Right-Wing Authoritarian Scale (Altemeyer, 2007). Although the full scale includes 22 items, we shortened the measure to 16 items due to space limitations in our survey. (For a list of the included items, please write to the corresponding author.) A sample item is “There are many radical, immoral people in our country today, who are trying to ruin it for their own godless purposes, whom the authorities should put out of action.” A 7-point Likert scale was used to elicit responses, offering options from *Strongly disagree* (1) to *Strongly agree* (7). The Cronbach’s alpha in the present dataset was found to be .95, indicating high internal consistency for the abridged version of the scale.

The second scale, aimed at assessing endorsement of rigid hierarchical structures, was the Social Dominance Orientation scale (Pratto et al., 1994). We used the full 16-item scale, which is commensurate in length with the shortened RWA scale used here. Sample items are “To get ahead in life, it is sometimes necessary to step on other groups” and “It’s OK if some groups have more of a chance in life than others.” A 7-point Likert scale was used to elicit degree of endorsement, running from *Very negative* (1) to *Very positive* (7). The Cronbach’s alpha in the present dataset was found to be .97, indicating high internal consistency.

#### Perceived Psychopathic Behaviors of the Candidates

We used items from the Psychopathy Checklist–Revised (PCL-R; Hare, 2012), which is composed of 20 items (e.g., “failure to accept responsibility for own actions”) and it is typically completed by a mental health professional who has access to comprehensive information about a target individual. To adapt the scale for our present purposes, we deleted three items that are specific to criminal evaluations (i.e., juvenile delinquency, revocation of condition release, and criminal versatility), and we expanded the three-point scale to a more sensitive 5-point Likert scale (1 = *Never*; 2 = *A little*; 3 = *Sometimes*; 4 = *Often*; 5 = *Very often*). We introduced the set of questions with this context: “Presidential candidates: Now we are going to ask you about your perceptions of the two main presidential candidates in the last election. Please tell us how much each person displays each of the characteristics displayed along the left side of the box.” Descriptors such as “many short-term relationships” were arrayed on the left, and five Likert–style options were displayed for Donald Trump and the same options separately for Hillary Clinton. In addition to the 17 psychopathy items (see Appendix), we also randomly inserted 10 positive filler items (e.g., “acts like a good role model” and “cares about others”). In the present study, the Cronbach’s alpha for Trump-rated psychopathic tendencies was .95 (.92 for Trump voters and .91 for Clinton voters), and the alpha for Clinton-rated psychopathic tendencies was .94 (.85 for Trump voters and .91 for Clinton voters).

#### Self-Reported Vote

At the end of the survey, we asked, “Who did you vote for in the 2016 Presidential Election? (Please remember that your answer is anonymous, so we ask for you to be honest.)” As alternatives, we offered *Donald Trump*, *Hillary Clinton*, *Other (third party candidate)*, *Prefer not to say*, and *Did not vote*. We only included individuals in the final dataset who selected one of the first two options.

### Procedure

Participants were recruited within the MTurk online survey website for a study named “political views about today’s society” in the two weeks following Donald Trump’s presidential inauguration on January 20, 2017. Demographic variables were assessed but no name or other identifying information was obtained. Completion of the entire survey took about 20–30 minutes, and participants were paid $3 for their time. Ethics approval was obtained from the first author’s host institution.

### Transparency and Openness

Data, copies of surveys, and the codebook for both studies are available at osf.io in a folder titled “Perceptions of psychopathy in U.S. presidential candidates” (se Jose, 2025) Data were analyzed using SPSS and Mplus for this report. This study's design and its analyses were not preregistered.

## Results: Study 1

### Descriptive Statistics

Means, standard deviations, and correlations for the two groups of voters are reported in Table 1.

**Table 1:** Means, Standard Deviations, and Correlations of the Key Variables Separately by Trump and Clinton Voters

Electoral Subset	SDO	RWA	Perceived psychopathy of Trump	Perceived psychopathy of Clinton	*M*	*SD*
Clinton Voters (*N* = 154)						
SDO		.61***	-.44***	.28***	1.78	0.98
RWA			-.57***	.22**	2.25	1.17
Perceived psychopathy of Trump				-.28***	4.24	0.69
Perceived psychopathy of Clinton					2.08	0.65
Trump Voters (*N* = 159)						
SDO		.44***	-.14~	.12	3.25	1.45
RWA			-.23**	.12	3.87	1.23
Perceived psychopathy of Trump				-.25***	2.79	0.82
Perceived psychopathy of Clinton					3.37	0.67

*Note*. SDO = social dominance orientation; RWA = right-wing authoritarianism; item ranges for both are 1–7. Psychopathy = perceived psychopathic tendencies, range from 1-5; *SD* = standard deviation.

∼*p* < .10. *p < .05. **p < .01. ***p < .001.

Less than 1% missingness was noted, Little’s chi-square test yielded *p* > .30, and EM imputation was used to obtain a full dataset. No outliers were noted. Means for self-reported SDO and RWA scores were higher for Trump voters compared with Clinton voters, consistent with expectation. Means for attributions of psychopathic tendencies for own vs. other candidate seem to be mirror images.

### Hypothesis 1: Was Trump Perceived as More Psychopathic Than Clinton?

To analyze the data for Hypothesis 1, we performed a repeated measures MANOVA in which attributions of psychopathic tendencies were the repeated measures across the two groups of voters. Further, we covaried out gender, ethnicity, highest schooling obtained, and age. A power analysis (Cohen, 1992) based on power at .80, an alpha level of .05, and an intention to identify medium effect sizes, suggested that a sample size of 125 would be needed. Our total sample of about 300 satisfied this initial basic criterion, but covarying out four covariates and planned analyses of interactions increased the recommended total sample to 325. Thus, our obtained sample fell slightly short of this more rigorous sample target. Caution is recommended for interpreting small effect size differences.

Contrary to prediction, we obtained a non-significant difference between the two candidates on perceived psychopathy, Wilk’s Λ = .998, *F*(1, 307) = 0.61, *p* = .436, $\eta_{p}^{2}$ = .002: collapsing across the two groups of voters, In addition, a non-significant effect for Voter was obtained, *F*(1, 307) = 2.71, *p* = .101, $\eta_{p}^{2}$ = .009, suggesting that Trump and Clinton voters attributed almost identical amounts of psychopathy, collapsing across the two candidates.

### Hypothesis 2: Did Voters Perceive Higher Psychopathic Behaviors in the Disfavored Candidate Than in Their Favored Candidate?

**Figure 1:**
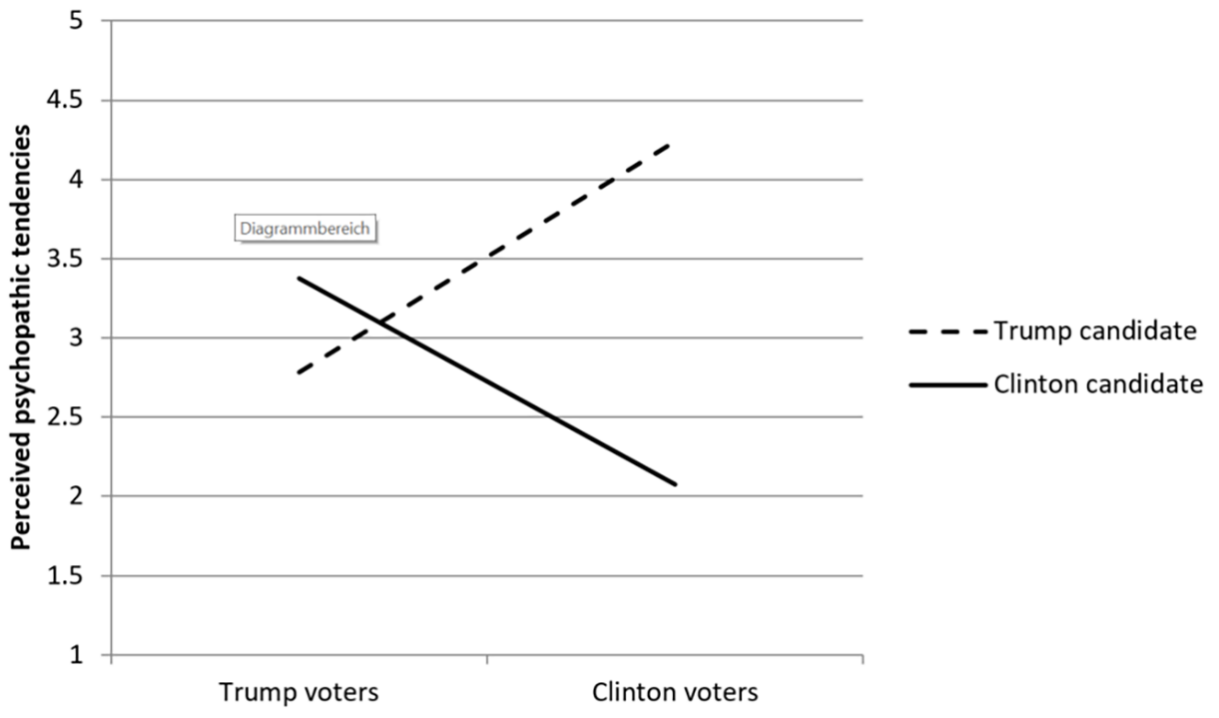
Perceived Psychopathic Behavioral Tendencies of Both Candidates by Voting Choice

By far and away, the largest effect size obtained in this analysis was the significant Candidate × Voter interaction, Wilk’s Λ = .405, *F*(1, 307) = 451.37, *p* < .001, $\eta_{p}^{2}$ = .60. Estimates of partial eta squared greater than .14 are considered “large” (Gray & Kinnear, 2012), so the effect size of this interaction can be considered to be extremely large. Figure 1 graphically depicts the interaction effect, and although it conforms to a classic cross-over interaction, the largest divergence occurred among Clinton voters considering the two candidates. Although both groups of voters rated their disfavored candidate as higher on psychopathic tendencies, Clinton voters’ perceptions more widely diverged than did Trump voters.

Simple effects analyses indicated that all four pairwise comparisons were significantly different. In sum, this pattern is consistent with Hypothesis 2, which suggested that voters would attribute psychopathic tendencies to the two candidates in a mirror image fashion: lower for their own candidate and higher for the opposing candidate.

### Hypothesis 3: How Would Authoritarian/Socially Dominant Beliefs Be Associated With Attributions of Psychopathic Tendencies and Vote Choice?

**Figure 2:**
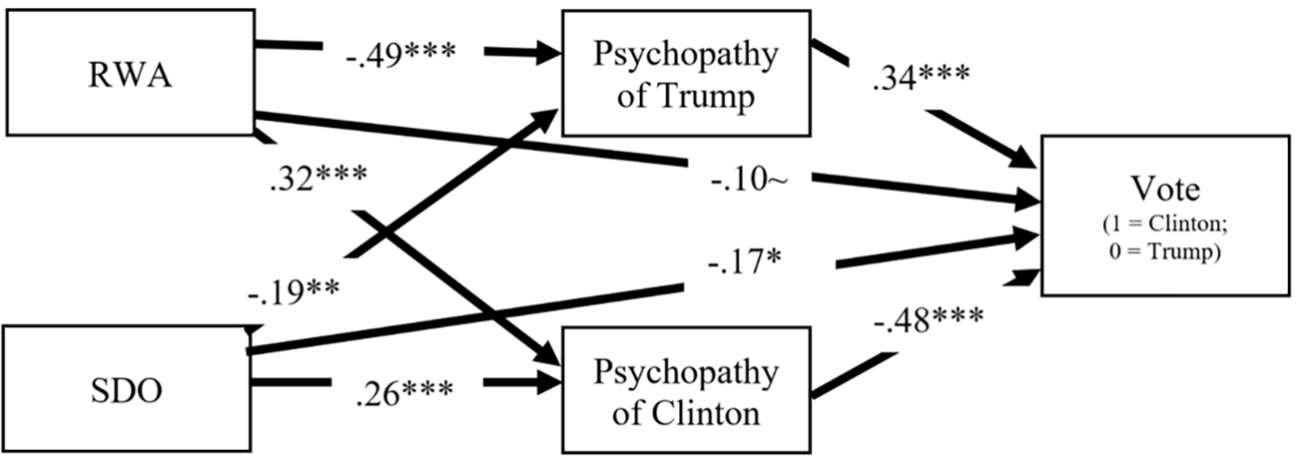
Path Model Including Background Authoritarian Beliefs, Perceptions of Psychopathic Tendencies of Candidates Trump and Clinton, and Voting Choice *Note*. RWA = right-wing authoritarianism; SDO = social dominance orientation; Psychopathy = perceived psychopathic tendencies; Vote = self-reported voting choice. ~*p* < .10. *p < .05. **p < .01. ***p < .001

A path model was constructed in Mplus (Muthén & Muthén, 1998–2015) in which background ideological beliefs (SDO and RWA) predicted perceptions of psychopathic tendencies of both candidates and these, in turn, predicted vote choice (see Figure 2). Since the dataset was concurrent, this mediation analysis illuminated amounts of shared variance among the three variables, not temporal or causal relationships discernible with experimental or longitudinal datasets (Jose, 2013). In such a case, sequential placement of variables in the model can be uncertain. We positioned these variables in this particular order for theoretical reasons (i.e., background personological variables were expected to explain variance in attributions of psychopathy to candidates, which, in turn, were expected to explain variance in vote choice behavior). To obtain a generalizable result, covariates of the model were gender, age, highest educational attainment, and ethnicity (not depicted in the figure). The fully saturated path model featured a dichotomous dependent variable in voting choice, so theta parameterization in Mplus (Muthén & Asparouhov, 2002) was used to accommodate this variable type.

The numerical values in Figure 2 are beta weights, and every path was significant in the expected direction, except for one path which was marginally significant. Several significant direct effects can be noted in the model: (1) high RWA and high SDO predicted higher perceptions of psychopathic tendencies in Clinton; (2) in contrast, high RWA and high SDO predicted *lower* perceptions of psychopathic tendencies in Trump; (3) higher perceptions of psychopathic tendencies in Trump and lower perceptions of psychopathic tendencies in Clinton predicted voting for Clinton (and the reverse pattern for voting for Trump); and (4) RWA and SDO weakly and directly predicted voting for Trump.

Four significant indirect effects were identified: 1) RWA to psychopathic tendencies in Trump to Vote: a*b = -.29, *SE* = .09, 95% CI [-.47, -.15], *p* < .001; 2) RWA to psychopathic tendencies in Clinton to Vote: a*b = -.26, SE = .08, 95% CI [-.44, -.13], *p* < .001; 3) SDO to psychopathic tendencies in Trump to Vote: a*b = -.11, SE = .05, 95% CI [-.23, -.03], *p* = .027; and 4) SDO to psychopathic tendencies in Clinton to Vote: a*b = -.21, SE = .08, 95% CI [-.38, -.08], *p* = .005. These indirect effect sizes fell between medium and large (Kenny, 2024). In sum, these findings supported Hypothesis 3: We found that voters reporting higher authoritarian beliefs perceived fewer mental health problems in Trump’s behavior while at the same time they perceived greater psychopathic tendencies in Clinton. In contrast, individuals reporting lower levels of authoritarian beliefs evidenced associations in the opposite direction.

## Method: Study 2

The goals of the second study were to replicate and extend the key findings from Study 1. The first three years of Trump’s presidency were marked by several key actions that may have contributed to increases in perceptions of psychopathy by voters: His reaction to the alt-right march in Charlottesville, his efforts to build a wall on the southern border, and his executive order banning people from six Muslim-majority countries from entering the U.S. We sought to determine the persistence of the relationships identified in Study 1 as well as entertain the possibility that these views might be more polarized after three years of Trump’s presidency.

We obtained a comparably sized sample of voters in the two weeks following Trump’s acquittal in his first impeachment trial on February 5, 2020. The same measures were used with a newly recruited sample of Trump and Clinton voters within a cohort study design. Although obtained at two different times of measurement, these data are not longitudinal, so we could not examine intraindividual change over time. Nevertheless, this attempt to replicate Study 1’s findings was considered to be valuable because the results would speak to the durability (and/or changeability) of perceptions of psychopathy for key political figures over time.

### Participants

As before, using the online survey tool MTurk, we recruited adults who had voted in the 2016 U.S. presidential election, and we obtained data from 149 individuals who reported that they had voted for Donald Trump and 148 individuals who reported that they had voted for Hillary Clinton. The sample included 147 males and 150 females, and this ratio was similar for Clinton and Trump voters in the sample, χ^2^(1) = 2.79, *p* > .10. The age range was 20 to 78 years, and the average age of respondents was 40.97 years (*SD* = 18.13). The age difference between Clinton and Trump voters was small, *t*(295) = 0.86, *p* = .589; Trump voters (*M* = 41.6 years) were slightly older than Clinton voters (*M* = 40.3 years).

## Results: Study 2

### Descriptive Statistics

Cronbach’s alphas of all key constructs for Study 2 were high and very similar to Study 1: SDO (α = .94), RWA (.94), perceived psychopathic traits of Trump (.96), and perceived psychopathic traits of Clinton (.94).

A chi-square test of gender yielded a significant difference between the two studies: χ^2^(1) = 4.59, *p* = .032, revealing that the male/female gender ratio was more equal for Study 2 (49%) than Study 1 (58%). A similar chi-square test of white vs. non-white ethnicity yielded a non-significant difference, χ^2^ = 1.63, *p* = .201. The sample of Study 2 was found to be older (*M* = 40.1) compared with Study 1 (*M* = 35.0), *t*(608) = 4.97, *p* < .001. A chi-square test of highest level of schooling yielded a significant difference: χ^2^(5) = 17.6, *p* = .003, revealing that Study 2 included fewer individuals with high school degrees and more individuals with professional degrees than Study 1. These results led us to covary out gender, age, and level of schooling in comparing mean levels and associations between the two samples in subsequent analyses.

Table 2 reports the correlations, means, and standard deviations of the four key variables divided by Trump vs. Clinton voters within Study 2.

**Table 2:** Means, Standard Deviations, and Correlations of the Key Variables Separately by Trump and Clinton Voters for Study 2

Electoral Subset	SDO	RWA	Perceived psychopathy of Trump	Perceived psychopathy of Clinton	*Mean*	*SD*
Clinton Voters (*N* = 148)						
SDO		.63***	-.52***	.47***	1.91	0.97
RWA			-.65***	.41***	2.24	1.07
Perceived psychopathy of Trump				-.47***	4.37	0.63
Perceived psychopathy of Clinton					2.12	0.72
Trump Voters (*N* = 149)						
SDO		.54***	-.34***	.32***	3.27	1.16
RWA			-.36**	.18*	3.90	1.06
Perceived psychopathy of Trump				-.07	2.97	0.92
Perceived psychopathy of Clinton					3.43	0.70

*Note*. SDO = social dominance orientation, range from 1–7; RWA = right-wing authoritarianism, range from 1–7; Psychopathy = perceived psychopathic tendencies, range from 1–5; *SD* = standard deviation.

*p < .05. **p < .01. ***p < .001.

### Replication of Findings From Study 1

We next analyzed the data from Study 2 alone; as before, we performed a repeated measures ANOVA in which attributions of psychopathic tendencies was the repeated measure across the two groups of voters. Further, we covaried out gender, ethnicity, highest schooling obtained, and age.

First, we obtained a significant (albeit small) difference between the two candidates for perceived psychopathy, Wilk’s Λ = .986, *F*(1, 291) = 4.17, *p* = .042, $\eta_{p}^{2}$ = .014. Collapsing across the two groups of voters, Trump was perceived, on average, in Study 2 to display higher psychopathic tendencies, *M* = 3.67, *SE* = .045, 95% CI [3.58, 3.76], than Clinton, *M* = 2.78, *SE* = .041, 95% CI [2.70, 2.86]. Thus, support for Hypothesis 1 was obtained, however statistical significance was barely achieved and the effect size was small.

As before, a non-significant effect for Voter was obtained, *F*(1, 291) = 0.63, *p* = .427, $\eta_{p}^{2}$ = .002, suggesting that Trump and Clinton voters attributed almost identical amounts of psychopathy, collapsing across the two candidates.

**Figure 3:**
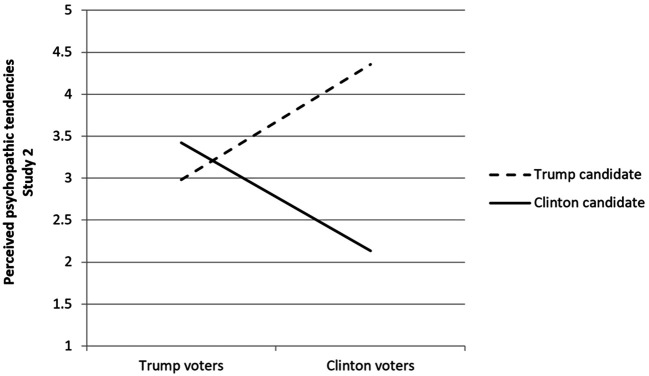
Study 2 Perceived Psychopathic Traits of Both Candidates by Voting Choice

Again, a very large effect size was obtained in this analysis for the Candidate × Voter interaction, Wilk’s Λ = .443, *F*(1, 291) = 365.40, *p* < .001, $\eta_{p}^{2}$ = .56. As with Study 1, this effect size was extremely large. The pattern of the interaction strongly supports Hypothesis 2. Figure 3 graphically depicts the interaction effect for Study 2, and it bears a remarkable similarity to the same graph generated for Study 1.

Next, to explicitly compare the ratings between Study 1 vs. Study 2, we performed a three-way mixed model MANOVA in which Study (1 vs. 2) was crossed with Voter, and the repeated measures factors were perceived psychopathic traits for the two candidates, and four demographic variables were entered as covariates. The main effect for Study yielded a significant difference, *F*(1, 602) = 11.45, *p* < .001, $\eta_{p}^{2}$ = .02 (albeit a small effect size), and it was accounted for by slightly higher average ratings of perceived psychopathic traits for Study 2, *M* = 3.23, 95% CI [3.18, 3.29], compared with Study 1, *M* = 3.11, 95% CI = [3.06, 3.16]. These means were determined after collapsing across candidates and voters, and covarying out demographic variables. Importantly, no interaction term involving Study yielded significance: Study × Voter (*p* = .73); Study × Candidate (*p* = .21); or Study × Candidate × Voter (*p* = .65). The single significant univariate result was a main effect for Study for perceived psychopathic traits for Trump, *F*(1, 602) = 7.91, *p* = .006, $\eta_{p}^{2}$ = .012. Notably, *both* Trump and Clinton voters (i.e., no significant three-way interaction was found) evidenced a small increase in these perceptions from the time of the election (*M* = 3.50) to the first impeachment (*M* = 3.69). A change of .19 unit constituted about 18% of the standard deviation for this variable and, interestingly, Trump voters increased slightly more than Clinton voters. Against the backdrop of overwhelming similarity in means for variables over time, the one key difference was a modest increase in the perceived psychopathy of Trump by both Clinton and Trump voters after three years of his presidency.

The final replication effort was to compare the path model findings of Study 1 with the same model constructed with Study 2 data. To statistically evaluate similarity, equality constraints were applied to all paths (direct paths as well as mediations) one by one, and all eight direct tests and four indirect tests yielded non-significance, *p*s > .05. Thus, Hypothesis 3 was supported in that perceptions of candidates’ psychopathy were found to significantly share variance with RWA/SDO scores as well as the decision to vote for Trump or Clinton. More broadly, this result, similar to the MANOVA findings, provides support for the proposition that these findings were almost universally replicated with a different set of participants at a different point in time.

## General Discussion

The question of political candidates’ fitness for office is an issue of paramount importance for society’s well-being because elected politicians consequentially ‘steer the ship of state.’ In the U.S., voters make the ultimate decision of whether a person is suitable for a particular office or not, and events over recent years have raised questions about how well voters discern critical qualifying and disqualifying characteristics in candidates. The present research project examined three key issues relevant to this issue. We first sought to determine whether voters generally would yield a significant difference in attributing more psychopathic traits to Trump than to Clinton. The initial data collection indicated a conclusive “No” and the second data collection after Trump’s first impeachment trial indicated “Weakly yes.” Given the effect size of the small difference in the second data collection and the sample sizes, we conclude that the evidence for this hypothesis was weak.

In the second case, we explored the hypothesis that Trump and Clinton voters would view their favored candidate differently from their disfavored candidate in terms of attributed psychopathy. The significant Voter × Candidate interaction that we found was extremely large, and it strongly supported our view that psychopathy would be attributed to the rejected candidate, but not to the favored candidate, and this pattern was apparent in opposite directions for both Trump and Clinton voters. In particular, Clinton voters perceived high levels of psychopathic behavior in Trump and low levels of psychopathic behavior in Clinton, whereas Trump voters evidenced the opposite pattern.

Next we sought to test the third hypothesis that background authoritarian beliefs by voters would be associated with attributions of psychopathy and voting behavior. The path model revealed that voters who reported high levels of authoritarian/social dominance beliefs were likely to perceive candidate Trump as low in psychopathy and candidate Clinton as high in psychopathy, which, then in turn, predicted for whom they voted. Highly authoritarian/SDO individuals tended to vote for Trump and against Clinton.

And fourth and last, a comparison of data obtained at Trump’s inauguration (Study 1) and after the first Trump impeachment trial (Study 2) evidenced remarkable stability of means and associations, but a small effect over time was noted in a general increased tendency to evaluate Trump as more psychopathic, possibly due to his performance in office up to that point.

### Linkages of Findings to the Literature on Lay Perceptions of Psychopathic Traits

Mental health literacy, as defined by Jorm (2000), is the skill and knowledge base of laypeople to accurately label instances of behavior as conforming to particular psychiatric syndromes. Little research evidence has been obtained on this issue, but the available evidence suggests that laypeople are poor at correctly labelling even well-known mental disorders such as depression and schizophrenia (Furnham et al., 2011; Jorm et al., 1997; Regier et al., 1988). Research is scarce on whether laypeople are able to correctly identify instances of psychopathic behavior (a type of personality disorder) and research is further lacking on whether voters are able to accurately identify psychopathic behavior in political candidates and officeholders. One significant contribution of the present work is that it broaches the question of how skilled laypeople are in the ability to identify disqualifying psychological characteristics of candidates. The present work does not tackle the issue of how accurate these perceptions were (we had no means to make this determination), but future work should explore that issue.

A notable finding in the study was that the cross-over statistical interaction depicted by Figure 1 revealed an almost mirror-image pattern of voters attributing absence of psychopathy in their preferred candidate at the same time as attributing presence of psychopathy in the rejected candidate. This dramatic and opposite bifurcation of views is consistent with ingroup vs. outgroup perceptions held by Democrats and Republicans in U.S. politics (Greene, 2004) and the phenomenon described by Wright and Tomlinson (2018), that political orientation profoundly shapes one’s attributions of personality to candidates. We did not assess political affiliations of voters in these data collections, but voter preferences in these data likely well represent voters’ political loyalties. The interaction pattern strongly suggests that voters see no flaws in their preferred candidate and see many disqualifying problems in their disfavored candidate.

However, the pattern was not perfectly symmetrical: Clinton voters more strongly attributed psychopathy to Trump and lack of psychopathy to their own candidate than did Trump voters. In other words, Clinton voters more strongly felt that their candidate lacked psychopathic traits relative to the opposing candidate. The widespread and intense incredulity and dismay of Clinton voters to Trump’s electoral victory is consistent with the highly polarized perceptions of psychopathic behavioral tendencies obtained in this study. Clinton voters were surprised and dismayed that the candidate they perceived as exceedingly unsuitable for the office of president was elected by the American public to the highest office in their country (DeGroot & Carmack, 2021; Teng, 2017).

### Linkages to the Literature on Associations Among Authoritarian/SDO Beliefs, Candidate Perceptions of Psychopathy, and Voting Choice

The set of results obtained in the path model may provide some tentative answers as to why so many voters chose to vote for Donald Trump. Our prediction was that individuals who held high levels of authoritarian/SDO beliefs would be likely to perceive Trump’s behaviors as consistent with the authoritarian mindset and thus view him more positively than Clinton (based on findings by Crowson & Brandes, 2017). In short, authoritarian-leaning voters attributed psychopathic traits, for example, dishonesty and a tendency to con other people, to Clinton and did not attribute these pernicious traits to Trump, whereas voters lacking in authoritarian beliefs attributed psychopathic traits to Trump and not to Clinton. And last, unsurprisingly, the path model showed that individuals who attributed high levels of psychopathy to Trump tended to vote for Clinton, and those individuals who attributed high levels of psychopathy to Clinton tended to vote for Trump.

The results of this study support the “beauty is in the eye of the beholder” theory of perceptions of political actors (Killian, 2023). Clinton voters were aghast at the stance of Trump voters explaining away the Access Hollywood recording concerning Trump’s claim of “pussy grabbing” as “locker room talk,” while at the same time many Trump voters wanted (and still want) Clinton to be jailed for perceived criminal mishandling of her e-mails. This extreme polarization of political views may be explained in part by the range of authoritarian beliefs held by voters. If a voter holds strong authoritarian/SDO views, Trump’s actions and words are not considered to be psychologically disturbed or psychopathic; instead, they are perceived as evidence of his strong leadership skills and socially dominant status. Thus, a voter who endorses authoritarian views is likely to view Trump’s treatment of minorities as legitimate and appropriate. Someone who rejects authoritarianism interprets Trump’s behavior differently, noticing the motives for self-aggrandizement and deception, imposition of power over other people, and joy in the misery of others (Lee, 2017).

These findings are consistent with Caprara and Zimbardo’s (2004) congruency model of political preference, which argues that “individual characteristics of voters, such as their traits and values, become decisive for political choice” (p. 581). Their findings reveal that people tend to vote for candidates whose personality traits accord with the ideology of their preferred political party. They also select politicians whose “traits match their own traits” (p. 581). The findings of the present study show that individuals who held authoritarian beliefs tended to vote for Donald Trump, the more overtly authoritarian candidate relative to Hillary Clinton, in agreement with Caprara and Zimbardo’s model. The theory of “ideology as motivated social cognition” similarly claims that personality characteristics inform and drive, to a substantial extent, political positions and choices (Carney et al., 2008; Fortunato et al., 2018; Jost, 2006; Jost & Amodio, 2012). Thus, the data suggest that an authoritarian ideology motivated Trump supporters to vote for him, and, in service of this behavior, attributed lower levels of aberrant behavior to him.

### Limitations of the Present Study and Future Directions

A significant caveat that we need to highlight is that the authors of the present paper have no authoritative evidence on the mental health status of Donald Trump or Hillary Clinton. However, it is relevant that empirical researchers, similar to us, have asked political experts, voters, and even politicians themselves about their perceptions of candidates (e.g., Dutton, 2016; Nai & Maier, 2018) But lacking objective, authoritative evidence, we could not, within the present study, ascertain whether voters *accurately* perceived the presence of psychopathic tendencies in the two U.S. presidential candidates.

Another significant limitation is that we do not know the direction of the influences of these perceptions and behavior over time, but, still, we ordered the three sets of variables within the path models to reflect a likely temporal sequencing. Beliefs in authoritarianism/SDO are stable belief systems partially based on personality, so they were placed as exogenous variables in the path model. Attributions of psychopathic characteristics were likely based upon the voters’ perceptions of the candidates’ behavior unfolding over months and years leading up to the day of the election when the voting choice was recorded. Longitudinal data would have provided a more nuanced picture of how these variables affected each other over time.

Another limitation in the present dataset is that party affiliation was not empirically assessed. Much research in political psychology has shown that party loyalty and identification exert considerable influence upon political perceptions and behavior (e.g., Folke & Rickne, 2020), and it would have been helpful to have this information to determine whether and how party affiliation interacts with authoritarian political beliefs in determining candidate preference. In particular, to what extent did Trump voters ignore or discount reports of his unsavory behavior out of party loyalty? At the same time, boundaries of political parties move over time to accommodate potential new members and new political leaders (Habersack, 2024). An illuminating future research direction would be to determine how party loyalty affects perceptions of candidates over time as critical events unfold.

In addition, future work should examine the mediators between authoritarian belief systems and attributions of psychopathy. A hypothesis worthy of further examination is to what extent do authoritarian voters publicly approve candidates who use extreme (and possibly criminal) behaviors to exert dominance over marginalized outgroups. Another useful direction would be to include measures of constructs antithetical to right-wing authoritarianism and social dominance orientation such as belief in democratic systems and racial equality (“wokeness”; Jose, 2024). And last, further investigation of the willingness of Trump supporters to excuse and dismiss his overtly antisocial behavior is needed. It is not clear at present whether they are uninformed, informed but consider this behavior as irrelevant, informed but consider his behavior as proof of his dominant and superior status, or something else. We would also like to extend the examination of this attributional bias to other politicians’ supporters to determine whether supporters across the full range of political affiliations show similar or dissimilar biases.

### Conclusions and Implications

The 2016 U.S. presidential election raised a number of important questions about how voters in the American democratic system consider candidates’ behavior and estimate future political performance of candidates. One such question is whether the average American voter possesses a sufficient knowledge base to discern significant psychological pathologies in candidates and uses those judgements to guide their choice among candidates. We need to improve our understanding of how background ideological beliefs, whether benign or malignant to the body politic, may influence perceptions of candidates and consequently lead individuals to cast their vote for one individual or another. The consequences of misperceiving psychopathic tendencies in candidates are potentially immense, and our democratic institutions need to identify and minimize such dangers.

## Supplementary Materials

For this article, the following Supplementary Materials are available:

- Data. (Jose, 2025)
- Codebook. (Jose, 2025)
- Study materials. (Jose, 2025)

## Data Availability

For this article, data, code, codebook and materials are available at Jose (2025).

## Appendix

### Psychopathy Items

1. Grandiose sense of self-worth
2. Lack of remorse or guilt
3. Superficial charm
4. Callous/lack of empathy
5. Pathological lying
6. Displays a need for stimulation
7. Behavior problems in childhood/adolescence
8. Promiscuous sexual behavior
9. Lack of realistic goals
10. Shallow affect (lack of authentic feelings)
11. Irresponsibility
12. Poor behavioral controls
13. Many short-term relationships
14. Failure to accept responsibility
15. Conning/manipulative
16. Impulsivity
17. Parasitic lifestyle (living off other people)
